# Do We Really Need Another Special Issue on NF-κB in Cancer and Inflammation?

**DOI:** 10.3390/cancers11121978

**Published:** 2019-12-09

**Authors:** Claudia Geismann, Alexander Arlt

**Affiliations:** Laboratory of Molecular Gastroenterology & Hepatology, Department of Internal Medicine I, UKSH-Campus Kiel, 24105 Kiel, Germany; cgeismann@email.uni-kiel.de

This series of 10 articles (four original articles, six reviews) is presented by international leaders in the field of NF-κB signaling in cancer and inflammation. While the reviews focus on the role of NF-κB signaling pathways in different types of solid tumors [1,2,3,4], B-cell lymphoma [5] and cancer stem cells [6], the four included original publications [7,8,9,10] address these aspects in single-cell analysis, complex animal models, and state of the art expression analysis.

Brenner and Bruserud [10] investigate the role of NF-κB in TLR signaling of acute myeloid leukemia. For the majority of a cohort of patients suffering from this very heterogeneous disease, the authors were able to show, that TLR4 is activated while TLR1/2 response is restricted to limited subgroups and correlate with prolonged survival, a higher NF-κB dependent basal cytokine expression, and a higher percentage of blasts in the population.

In a sophisticated approach, Riedlinger et al. [7] analyze the effects of the widely used chemotherapeutic drugs and topoisomerase inhibitors campotothecin, topotecan, and SN-38 on TNFα-induced gene expression. Using the RNA-seq method, the authors were elegantly able to show that inhibition of topoisomerase 1 but not of topoisomerase 2 activity suppressed the vast majority of TNFα/NF-κB-controlled genes. These effects are fully reversible and preferentially affect long genes. In summary, topoisomerase I inhibitors might be favourable in disease where pro-inflammatory cytokines (please refer to the review of Geismann et al. [3]) act as tumor promotors but might also explain the increased infection risk occurring in tumor patients treated with these agents. This aspect of NF-κB regulation in the context of proinflammatory cytokines is addressed in another paper of this special issue [8]. Since the interaction of tumor cells with proinflammatory cells is a hallmark of many cancers, the authors aim to study signaling pathways at the single-cell level with high precision, sensitivity, and spatial resolution. By using proximity ligation assays (PLAs) alone or combined with immunostaining for p65 or with NFKBIA single-molecule mRNA-FISH, they were able to show the heterogeneity of the NF-κB response in individual cells. In addition, they confirmed an interaction between NF-κB p65 and the P-body component DCP1a. This new p65 interactor contributes to efficient p65 NF-κB nuclear translocation. In the fourth original article, the role of NEMO in hepatocarcinogenesis is analysed in state of the art animal models [9]. By using mice lacking all three IKK subunits (IKKα, IKKβ, and NEMO) in liver parenchymal cells and comparing these mice with mice lacking both catalytic subunits (IKKα/β^LPC-KO^), the authors were able to describe I-κB-Kinase-independent functions of the regulatory subunit NEMO. In this context, a specific role in controlling programmed cell death of liver parenchymal cells, proliferation, cholestasis, and hepatocarcinogenesis is attributed to free NEMO molecules. Despite this IKK/NF-κB independent function of NEMO in hepatocellular carcinogenesis, there is overwhelming evidence of a central role of the NF-κB signaling module in nearly all aspects of liver cancer, as reviewed by Czauderna et al. [4]. The authors highlight the role of chronic inflammation-driven NF-κB signaling for the development of liver cancer but also raise the concern that absence of NF-κB in differentiated liver cells can also lead to HCC development. Thus, any NF-κB targeting approach can have beneficial but also disastrous effect. In the review the complexity of NF-κB signaling in primary liver and non-parenchymal cells is discussed, addressing this issue and leading to the conclusion that therapeutic interventions should particularly focus on immunotherapeutic approaches.

In addition to the discussion of HCC, the special issue includes a review of ovarian cancer [5]. For this cancer entity, many alterations in the NF-κB pathway are reported to lead to chemoresistance, cancer stem cell maintenance and self-renewal, metastasis and immune evasion. The aspect of cancer stem cells is extensively discussed in the manuscript by Kaltschmidt et al., focusing on this emerging field of research [6]. In ovarian cancer, NF-κB signaling can lead to an immune-evasive environment, accompanied by an enhanced tumor-promoting immune cell infiltration. In line with this central role of integrating the immune system in the development of solid cancer, the review of Geismann et al. [3] focus on the role of NF-κB controlled chemokine signaling in pancreatic cancer. These either autocrine- or paracrine-acting chemokines can operate in a tumor-promoting or -inhibiting fashion, making a context-specific approach for the consideration of this signalling module in cancer therapy inevitable. In this context, for chemokines like CCL20 [11] and CX3CL1 [12], an onco immuno crosstalk that leads to a NF-κB-dependent resistance against death receptor signaling is described. In addition to the Rel-subunit specific function in pancreatic cancer [1], the two reviews on pancreatic cancer of this series confirm a central role of NF-κB in many aspects of this lethal tumor entity. The subunit-specific role in cancer is addressed in another review [5] for B cell lymphoma. In this disease, c-Rel is tightly regulated at multiple levels but alterations of these regulatory pathways as well as gene mutations and modifications in co-regulators like BCL11A and REL are frequently reported. Thus, in contrast to other tumors, c-Rel has a central role and is the only member of the Rel-family that can malignantly transform lymphoid cells in vitro.

In conclusion, this series of unique articles represents a collaborative, international effort to address all aspects of NF-κB signaling in cancer. 

In the coming years, more work is needed to decipher the subunit-specific role of, but also the effects of, an altered NF-κB signaling pathway in the tumor cells with regard to the role of anti-stroma therapy, chemoresistance, and immunotherapy. 
